# Supplementary material for: Potential facilitators and inhibitors to the implementation and sustainability of the community-based tuberculosis care interventions. A case study from Moshupa, Botswana
Source: PLoS One. 2023 Aug 10;18(8):e0290010. doi: 10.1371/journal.pone.0290010 (PMC10414663; doi:10.1371/journal.pone.0290010)
Supplement: S1 Table — (DOCX) [file pone.0290010.s002.docx]

S1 Table: Operational definitions of measurement constructs in the Community Health Worker Performance Measurement Framework [7]

| **Inputs** | | | | |
| --- | --- | --- | --- | --- |
|  | **Indicator domains** | | **Definition/explanation** | |
| 1. | Policies | | National-level policies that support the development and deployment of CHW programs | |
|  | CHW selection | | Age, education and other policy-supported requirements for being eligible to become a CHW are listed | |
|  | CHW tasks/workload | | Description of the role and tasks to be performed by a CHW from the community, CHW and health systems perspectives | |
| 2. | Stakeholders | | Engagement with the Ministry, agencies and other stakeholders to support the CHW program | |
| 3. | Logistics | | Provisions, material and technological to support CHW functions. | |
|  | Transportation | | Provisions, either monetary (fare for busses) or physical (bicycle) for CHWs to physically access target population | |
|  | Commodities | | Required equipment, medicines and supplies to deliver services, as well as resources such as job aids to support the quality of services | |
| 4. | Funding | | Level of government/donor and other stakeholder investments in CHW programs in country | |
| 5. | Information management systems | | Support for CHW to document home visits including community-based health information systems, report visit related data to the health system and link it to an assessment of CHW performance | |
| **Programmatic processes** | | | | |
|  | **Indicator domains** | | **Definition/explanation** | |
| 1. | Supportive systems | | Structural processes that influence CHW functions at various levels of the health system (facility/local/sub-national/national) | |
| A. | Supervision | | Consistent and continued support for problem solving service delivery and skill.  development, including evaluation and supportive feedback on the work performed by the CHW in a set period | |
| 2. | CHW development | |  | |
| A. | Recruitment | | How and from where a community health worker is identified, selected, and assigned to a community | |
| B. | Training | | Training is provided to the CHW to prepare for his/her role in service delivery and ensure s/he has the necessary skills to provide safe and quality care. | |
| C. | Incentives | | Includes financial incentives such as salaries and bonuses, and non-financial incentives such as training, recognition, uniforms and other opportunities for advancement | |
| **Community health workers performance outputs-Individual level** | | | | |
|  | **Indicator domain** | | **Definition/explanation** | |
| 3. | CHW competency | | Degree to which CHW has the knowledge and skills necessary to carry out the assigned tasks | |
| A. | | Service quality | | Adherence to standards and procedures (counseling, health promotion, treatment accuracy) |
| B. | | Data reporting | | Regularity and completeness of CHW reports on the services they provide at the community level |
| 4. | | CHW well-being | | The overall well-being of the CHW may be seen as a measure of effectiveness of the system that supports the CHW program |
| A. | | Motivation | | An individual’s degree of willingness to exert and maintain effort on assigned tasks; a CHW’s confidence, belief in his/her ability to produce a desired result |
| B. | | Job satisfaction | | Degree to which CHWs derive personal satisfaction from serving the community and providing services |
| C. | | Attrition/retention | | The rate at which practicing CHWs resign, retire or abandon their positions |
| **Community health workers performance outputs-Individual level** | | | | |
|  | | **Indicator domain** | | **Definition/explanation** |
| 5. | | Community access | | Delivery of CH services in a timely manner within the client’s home/community OR clients’ physical/social access to CHW service |
| A. | | Use of services | | Clients are routinely seeking and using promotional, preventive and curative services that CHWs offer |
| 6. | | Community-centered care | | Community involvement, experience, and perceptions of services provided by CHWs |
| A. | | Empowerment | | Both individuals and communities participate actively in community health activities |
| B. | | Experience of care | | Clients’ experience of services delivered by CHWs, including respectful care, and clients’ perception of quality of care. |
| C. | | Credibility/trust of CHW | | Degree to which clients consider the services provided by CHWs to be credible and reliable |
