# Supplementary material for: Potential facilitators and inhibitors to the implementation and sustainability of the community-based tuberculosis care interventions. A case study from Moshupa, Botswana
Source: PLoS One. 2023 Aug 10;18(8):e0290010. doi: 10.1371/journal.pone.0290010 (PMC10414663; doi:10.1371/journal.pone.0290010)
Supplement: S2 Table — (DOCX) [file pone.0290010.s003.docx]

S2 Table: Sociodemographic attributes /characteristics of the respondents/participants from Moshupa, Botswana

| **Characteristics/variable** | **Frequency(n)** | **Percentage (%)** |
| --- | --- | --- |
|  |  |  |
| **Sex** |  |  |
| Male | 11 | 27.5 |
| Female | 29 | 72.5 |
| **Age groups** | | |
| 21–30 years old | 5 | 12.5 |
| 31–40 years old | 8 | 20 |
| 41–50 years old | 12 | 30 |
| 51-60 years old | 9 | 22.5 |
| 61-70 years old | 5 | 12.5 |
| ≥ 70+ | 1 | 2.5 |
| **Profession / responsibility/ Status of participant** | | |
| Head of DHMT (Medical officer) | 1 | 2.5 |
| Head of preventative | 1 | 2.5 |
| District TB coordinator | 1 | 2.5 |
| Nurse | 5 | 15 |
| TB screener | 1 | 2.5 |
| Data support officer | 1 | 2.5 |
| Village/tribal Headman | 4 | 10 |
| Community elders | 4 | 10 |
| TB treatment supporters | 22 | 55 |
| **Professional experience on TB matters (years)** | | |
| 1–3 years | 10 | 25 |
| 4–6 years | 3 | 7.5 |
| **>** 7 years | 27 | 67.5 |
| **Highest Level of Education** | | |
| Non-formal | 3 | 7.5 |
| Primary | 11 | 27.5 |
| Junior secondary | 9 | 22.5 |
| Senior secondary | 6 | 15 |
| Tertiary | 11 | 27.5 |
