# Supplementary material for: Potential facilitators and inhibitors to the implementation and sustainability of the community-based tuberculosis care interventions. A case study from Moshupa, Botswana
Source: PLoS One. 2023 Aug 10;18(8):e0290010. doi: 10.1371/journal.pone.0290010 (PMC10414663; doi:10.1371/journal.pone.0290010)
Supplement: S3 Table — (DOCX) [file pone.0290010.s004.docx]

S3 Table: Selected patient interview quotes categorized by the framework levels and barriers to and potential facilitators to the implementation and sustainability of the community-based tuberculosis care interventions.

| **Level** | **Categories** | **sub-categories** | **Facilitator** | **Barrier** | **Sample quotations** |
| --- | --- | --- | --- | --- | --- |
| Input | Policies | CHW selection |  | X | *“The SOP’s and policy are good but do not capture age and educational requirements for eligibility to be a treatment supporter, a younger treatment supporter may seem awkwardness to a patient posing challenge to DOT supervision whereas educational level governs the ability for one to read drugs and dosages thus influencing DOT and documentation”- (IDI, Nurse, Moshupa health post)* |
|  |  | CHW tasks | X |  | *“The facilities had national TB program policies and SOPs with description of the role and tasks to be performed by community health workers, treatment supporters and health systems perspectives were clearly documented,” – (TB clinic Observation 14th April 2022)* |
|  | Funding |  |  | X | *“The DHMT relies on government budget to provide for TB services which is often inadequate to fulfill the program activities thus making it difficult to design the program long-term maintenance and monitoring plans with confidence.” – (IDI, TB coordinator)*  *“The programme needs more support but there is a challenge of lack of funds to procure all resources needed.”- (IDI, Medical officer)* |
|  | Information management systems |  |  | X | *“We do not have any framework of software that facilitates the collection, storage, organization, and distribution of information. Instead, we use patient cards and file them as hard copies, report visit related data is given subjectively to the focal person or coordinator for action.” – (SSI, Treatment supporter, Mma-Seetsele clinic)*  *“Patients’ information was stored in paper files with some paper prints no longer visible, some torn”- (TB Registers Observation 13th April 2022)* |
|  | Stakeholders |  | X |  | *“The Moshupa village has partnered with (Bummhi) in provision of technical support including patient follow-up. The partnership partially counterbalances TB staff inadequacy in the district, which reduces TB-related morbidity and mortality”. -(IDI, TB Coordinator).*  *“Even though epidemic control has not yet been achieved, key measurements, such as numbers of people developing TB and numbers of people with TB-HIV co-infection have improved over the years”. – (IDI, TB focal person)* |
|  | Logistics | Transportation |  | X | *“We do encounter transport issues in our line of duty trying to assist our patients, only if we could be assisted with some transport money to board a taxi to collect patient medications and transport them for their reviews would help.” – (SSI, Treatment supporter, Moshupa SDA)*  *“Sometimes resources are limited for example lack transport to access TB patients at their homesteads thus impacting negatively on patient management, maybe if our government can consider reimbursement to treatment supporters so that whenever we don’t have transport, they can board taxis to bring the patient to health facility as per necessity.” – (IDI, Nurse, Moshupa health post)* |
|  |  | Commodities (equipment, medicines and supplies) |  | X | *“There is shortage of sputum induction machines, shortage of medications and there is also shortage of materials e.g., files and cards, thus we lose patient information.” – (IDI, TB screener, BUMMHI)*  *“Sometimes resources are limited, for example TB cards are often not enough, if we can ensure availability of such commodities we can be able to improve TB management.” – (IDI, Nurse, Mma-Seetsele clinic)*  *“We do encounter a shortage of medication at times and some tests are often omitted because of lack of machines to conduct those tests.” - (FGD with Community leader)* |
| **Level** | **Categories** | **sub-categories** | **Facilitator** | **Barrier** | **Sample quotations** |
| Programmatic processes | Supportive systems | Supervision |  | X | *“There is one TB focal person who also works on shift in outpatient department, when she is not there the program services suffer including supervision of community health worker as there will be no one to oversee the program with other unit staff members engaged with their assignments”. – IDI, Data support officer BUMMHI*  *“In our facility, workers are willing to be involved in TB care but there is no support/supervision from DHMT and TB coordinator, this lack of supervision leads to poor morale thus hesitancy to give their all in TB and its management.” – IDI, Nurse Moshupa health Post* |
|  | CHW development | Recruitment |  | X | *“Facility staff recruitment in TB care, e.g. TB focal person is not based on interest but rather unit task allocation potentially leading to unmotivated TB care employees who in turn do very little in delivery of TB services thus impacting the program negatively.” -IDI, Nurse Moshupa health post*  *“Treatment supporter selection is based on patient preferences, no literacy nor age requirements or other engagements consideration eg work elsewhere, this often leads to supporters leaving patients with medication to take on their own.” -SSI, Treatment supporter, Moshupa health post* |
|  |  | Training |  | X | *“The TB training is limited due to funds unavailability and staff that has been trained not orientating the new staff. This hampers TB progress to national strategy targets” -IDI, TB coordinator.*  *“I was never trained nor given proper coaching concerning the supervision of TB patients, I just self-taught myself on how a TB patient is taken care of.” – SSI, Treatment supporter* *Moshupa SDA* |
|  |  | Incentives |  | X | *“My wish is that we can be assisted with food because the socio-economic status is often low, and we don’t have money since we not working anywhere.” – “SSI, Treatment supporter, Mma-Seetsele clinic.*  *There is lack of incentives for treatment supporters hence it becomes difficult to enroll patients who stays alone.” -TB coordinator.*  *“Incentives like monetary allowances to treatment supporters can help motivate treatment supporters thus increase cure rates and reduce stigma to TB patients.” – IDI, Nurse Mma-Seetsele clinic* |
| **Level** | **Categories** | **sub-categories** | **Facilitator** | **Barrier** | **Sample quotations** |
| **Community health worker performance outputs-Individual level** | CHW competency | Service quality |  | X | *“Family members (Adherence buddy) tend to leave clients alone with medication without monitoring them hence clients defaulting treatment.” -IDI, Nurse Moshupa health post”*  *“I would explain to the patient how to take medication, because I would leave them with him to take when I’m not there because he knew the tablets.” – IDI, Treatment supporter, Mma-Seetsele clinic* |
|  |  | Data reporting |  | X | *“There is poor coordination between the doctor and the TB focal person as patients’ information is mostly not comprehensively documented on patients cards when the patient is initiated by the doctors e.g. contacts often not captured and the final treatment outcome is most of the time absent in the registries,” - IDI, Data support officer, BUMMHI*  *“TB registers and patient facility cards had missing/incomplete data in almost all facilities eg monthly compliance with treatment, sputum smear results and treatment outcomes data missing”- Registers Observation,13^th^ April 2022* |
|  | CHW well-being | Motivation/Job satisfaction |  | X | *“I wish to be the change agent and improve TB patients’ quality of lives in our community however being a treatment supporter is a lot of unpaid work, worse than that there is less support from family and health facility making work even more difficult therefore I don’t think I can risk myself when im not happy.”-SSI, Treatment supporter, Moshupa health post.* |
|  |  | Retention |  | X | “*Treatment supporters are no longer active, with a smaller number of staff trained on TB case management transferred out of the DHMT, and resigning treatment supporter thus crippling the program.” -IDI, Nurse Moshupa SDA* |
| **Community health worker performance outputs-community level** | Community access | Use of services |  | X | *“CTBC uptake is very low mainly due to patients not being interested in the program and health care workers not educating and counselling clients thoroughly on the program.” – (IDI, TB coordinator)*  *“Care givers are not co-operating with patients and health care workers as they often do not take medication well on time for their patients.)- (IDI, TB screener, BUMMHI)*  *“There is poor community involvement as most clients(patients) and care givers are reluctant to enroll on community TB care.” – (IDI, Medical officer)* |
|  | Community-centered care | Empowerment | X |  | *“The community was consulted, I remember I was in village development committees (VDC) then when the health education assistants were moving around the community taking care of patients, and the program has no conflict with the community sociocultural practices or believes”. - (FGD with Community leader)*  *“The community was consulted, I was one of those who were in the red cross, we would take medications on behalf of patients who were not able to carry out their daily activities, and deliver them at their homes” - (FGD with Community leader)*  *“The target population and community leaders are engaged in implementation, but more can still be done by involving them in development of the framework or implementation model”- (IDI, TB coordinator)* |
|  |  | Credibility |  | X | *“Some families felt we came to assess how they live or judge them when we go on family visit and that created some challenge in delivering the TB services”- (IDI, TB screener, BUMMHI))*  *“Patients preferred nurses over us as the NGOs and doubted the quality of service we can provide.” – (FGD with Community leader)* |
