# Supplementary material for: Potential facilitators and inhibitors to the implementation and sustainability of the community-based tuberculosis care interventions. A case study from Moshupa, Botswana
Source: PLoS One. 2023 Aug 10;18(8):e0290010. doi: 10.1371/journal.pone.0290010 (PMC10414663; doi:10.1371/journal.pone.0290010)
Supplement: Attachment — Submitted filename: Response to reviewers.docx [file pone.0290010.s006.docx]

The following are the editors comments/recommended changes to the Manuscript, authors responses and page number where changes were made presented in Tabular form.

| ***Editor’s comments/Recommended Changes*** | ***Authors Response*** | ***Changes Page Number*** |
| --- | --- | --- |
| **Table of content**   1. Please ensure that your manuscript meets PLOS ONE's style requirements, including those for file naming. | Recommendation done | Page 3-5  9-18 |
| 2. Please include a complete copy of PLOS’ questionnaire on inclusivity in global research in your revised manuscript. | Recommendation incorporated on the manuscript, Questionnaire included among supporting document | 8 |
| 3. Thank you for stating the following financial disclosure:  "The author(s) received no specific funding for this work."   At this time, please address the following queries:  a) Please clarify the sources of funding (financial or material support) for your study. List the grants or organizations that supported your study, including funding received from your institution.  b) State what role the funders took in the study. If the funders had no role in your study, please state: “The funders had no role in study design, data collection and analysis, decision to publish, or preparation of the manuscript.” c) If any authors received a salary from any of your funders, please state which authors and which funders. d) If you did not receive any funding for this study, please state: “The authors received no specific funding for this work.”  Please include your amended statements within your cover letter; we will change the online submission form on your behalf. | Recommendation incorporated and highlighted in yellow on the cover page | Cover page |
| 4. We note that you have stated that you will provide repository information for your data at acceptance. Should your manuscript be accepted for publication, we will hold it until you provide the relevant accession numbers or DOIs necessary to access your data. If you wish to make changes to your Data Availability statement, please describe these changes in your cover letter and we will update your Data Availability statement to reflect the information you provide | Data availability changed from available on request to data available within the article or its supplementary materials, change highlighted in yellow on the cover page and manuscript. | Page 34 of the manuscript  and cover letter |
| 5. our ethics statement should only appear in the Methods section of your manuscript. If your ethics statement is written in any section besides the Methods, please delete it from any other section. | Ethics statement deleted from the ending section of the manuscript |  |
| 6. We note that Figure S2 in your submission contain map image which may be copyrighted. All PLOS content is published under the Creative Commons Attribution License (CC BY 4.0), which means that the manuscript, images, and Supporting Information files will be freely available online, and any third party is permitted to access, download, copy, distribute, and use these materials in any way, even commercially, with proper attribution. For these reasons, we cannot publish previously copyrighted maps or satellite images created using proprietary data, such as Google software (Google Maps, Street View, and Earth). For more information, see our copyright guidelines: http://journals.plos.org/plosone/s/licenses-and-copyright.   We require you to either (1) present written permission from the copyright holder to publish these figures specifically under the CC BY 4.0 license, or (2) remove the figures from your submission:   a. You may seek permission from the original copyright holder of Figure S2 to publish the content specifically under the CC BY 4.0 license.     We recommend that you contact the original copyright holder with the Content Permission Form (http://journals.plos.org/plosone/s/file?id=7c09/content-permission-form.pdf) and the following text: “I request permission for the open-access journal PLOS ONE to publish XXX under the Creative Commons Attribution License (CCAL) CC BY 4.0 (http://creativecommons.org/licenses/by/4.0/). Please be aware that this license allows unrestricted use and distribution, even commercially, by third parties. Please reply and provide explicit written permission to publish XXX under a CC BY license and complete the attached form.”   Please upload the completed Content Permission Form or other proof of granted permissions as an ""Other"" file with your submission.   In the figure caption of the copyrighted figure, please include the following text: “Reprinted from [ref] under a CC BY license, with permission from [name of publisher], original copyright [original copyright year].”   b. If you are unable to obtain permission from the original copyright holder to publish these figures under the CC BY 4.0 license or if the copyright holder’s requirements are incompatible with the CC BY 4.0 license, please either i) remove the figure or ii) supply a replacement figure that complies with the CC BY 4.0 license. Please check copyright information on all replacement figures and update the figure caption with source information. If applicable, please specify in the figure caption text when a figure is similar but not identical to the original image and is therefore for illustrative purposes only | Figure S2 removed from the submission. | Caption on page three deleted  Also deleted on supporting document |
| 7. Please include captions for your Supporting Information files at the end of your manuscript, and update any in-text citations to match accordingly.. | Change incorporated | Page 34 |

The following are the reviewer’s comments/recommended changes to the Manuscript, authors responses and page number where changes were made presented in Tabular form.

| ***Reviewer’s comments/Recommended Changes*** | ***Authors Response*** | ***Changes Page Number*** |
| --- | --- | --- |
| Reviewer 1   1. The description of the methods section is not detailed enough to permit replication of the study without recourse to the authors. The design is described in a maze of imprecise lexicon –“Interpretive 7 paradigm based on relativist ontology and subjectivist epistemology along with adductive research”. This should be revised. As nebulous are these are, there is very little reflection of their application in the analysis and reporting of the study. | Justification for sampling captured, and conceptual framework table inserted on text to explain which variables are under study and what they asses. | Page 5-7 |
| 2. Important details like who conducted the interviews, where they were conducted, the nature of interview guide, how was the FGD organized? Was it one FGD with 8 community leaders? | Interviews were conducted by Principal investigator G.A.S alongside a trained research assistant, Focused group discussion and observation was done by lead author only.There was only one focused group discussion conducted.  The organisation of FGD was not captured for fear of manuscript word ballooning, however, this is how it was organised;   - Recruit and scheduling of participants - Have everyone introduce themselves - Get consent and start the discussion - Seek equal representation from the group - End the meeting in a reasonable amount of time - Analyze and incorporate feedback | Page 6-7 |
| 3. It is reported that clinic-based observations were conducted. There is however no information on how they were conducted. By whom? When and how? Was it with a standard tool? How was analysis of that data conducted?   Other concerns | Observation information beefed up to encompass the tool type and data collector | Page 6 |
| 4. The descriptive results in the opening paragraph of results is not comprehensive enough. The authors need to detail out the overall picture before going into the description by gender and age. And for each of the approaches to data collection, the authors need to be detailed in the characteristics of the participants | Recommendation incorporated, rephrased | Page 9-10 |
| 5. The results should also present a triangulation of information and not just a topic by topic representation with quotes. It is out of the triangulation of information that new understanding or theory emerges to add something new to the literature. | Data presentation revisited and presented in a manner that depict data triangulation. | Page 18-28 |
| 6. While it is well to present the Botswana program as having failed and needing to be researched, it is important for evidence to that effect to be presented. The authors need to provide data to back the claim of “low treatment outcome (this statement is unclear)” and “high case notification”? There should also be an attempt to link the above shortcomings to the delivery of Community TB Care. | Statement amended to;  However, despite the proven effectiveness of these low-cost strategies in controlling TB, Moshupa still experiences high TB incidence rates with low successful treatment outcomes compared to the national and WHO target. | Page 2 |
| Reviewers 2.  **General comments**   1. Is Moshupa a community, district? This is very confusing. | Moshupa is a village located in Southern District. | Amended in page 1 |
| 2. Appropriate or standard referencing style should be used, authors place the references after full stops.  - Line 371 and in several other sentences authors need to how they apply the references - Line 380 and several others: authors need to check the closeness of reference to words, no space etc. | Referencing amended, references put before the full stop throughout the paper.  Spacing between reference and words adjusted throughout the entire paper | Entire document |
| 3. The entire manuscript needs some editing to improve the language quality. The following are only but a few examples:  - Sometimes articles are missing, singular and plural tenses are not respected eg (line 398 “lack of incentives was frequently noted barriers to” - Some of the sentences need rephrasing eg line 400” “The indirect cost associated with the traveling, food and accessories were often pronounced.”; lines 400-402: … in Zambia(17) Malawi(31)(32) and Nigeria(33) which reported lack of incentives to affect motivation, retention of CHWs and slows the rate of program implementation. | Entire manuscript edited, captured line numbers also rephrased.  Incentives changed to singular  Rephrasing done | Page 31  Page 31 |
| 4. Very little literature has been explored in the introduction and discussion sections | Majority of the introduction literature was cited from the WHO and MOH(Botswana) documents hence why we used original sources mostly and some other sources equalling 8 in total.  On literature review we used 26 articles. |  |
| **Abstract**   5. Check the use of tenses such as singular and plural in the abstract, eg “Initiative employees for in-depth interview and 8 community leaders for focus group discussions”  - “the HIV negatives and 62/100 000 among the HIV positive” - Check parallel structure in sentences eg. Line 52 “decentralizing TB care beyond health facilities and harness the” - Check the use of capitals and small letters eg: Line 53 “The Botswana National Tuberculosis and leprosy Program (BNTP)” | Corrected with plural tense used for interviews in abstract  Corrected with singular tense used for HIV negative  Entire document re-edited, thus parallel structure identified addressed.  Leprosy was not sentence cased, it has been sentence/word cased | Page ii  Page 1  Page 1-2  Page 2 |
| **Introduction**   6. Authors should provide a brief description of the current state of TB policy or guidelines for implementation in the introduction. Later in the results, they can use real data to explain how the policy is actually being implemented, if this was part of their objective for the study. 7. There is no conceptual framework yet. The authors need to present a detailed well explained conceptual framework. The framework should explain the key concepts and how they were applied in the study. | TB guideline for implementation brief description captured  Conceptual framework table inserted on text detailing what the variables assessed | Page 2  Page 3-5 |
| Methods   7. Setting:  - No justification for the selection of Moshupa village.  8. Study design and population  - Justification for each group of participants selected is missing - What kind of information did each category of IDI/FGD guide seek to solicit? How many categories of IDIs/FGDs were developed and for which categories? - What do authors consider as groups? - Line 104: What does "self-developed mean" mean?  9. Procedures  - Who collected the data? | Selection justification provided. also captured in page 1  Justifications provided.  Type of information solicited inputted and the number of categories  Self-developed changed to researchers developed tools  Data was collected by Principal investigator G.A.S alongside a trained research assistant. | Page 5  Page 6-7  Page 6-7  Page 7  Page6- 7 |
| 10. Data analysis  - Nvivo version? - How were texts coded? Manual or with the support of Nvivo? - Were the notes also transferred to Nvivo for coding and analyses? - Who analyzed the data? - Kindly explain further the following statement: “Quality was ensured through joint assessment of the sampling approach and ongoing review of transcripts to explore areas for further probing" | Nvivo version 12 was used, ,notes were transferred into Nvivo for coding and analyses by principal investigator G.A.S  An extension to the statement added as follows;  was ensured through a joint assessment(Researchers) of the sampling approach and ongoing review of transcripts to explore areas for further probing thus maximizing the quality of the data collected | Page 7  Page 7 |
| 11. Results   Sociodemographic attributes of the respondents   - This is supposed to be a purely qualitative paper. Lines 85-90 in the methodology section report on very diverse groups of respondents who play different roles and have different experiences. How were these diverse groups lumped into one category for statistical analysis, what is the implication for the study itself and the results presented? - This study should remain a qualitative study, authors should not attempt to confuse the study. Thus, authors should take out the statistics and present a write up and table displaying the different categories and the background characteristics that is relevant to this study such as age, education, occupation/designation among others.  12. Qualitative findings  - Line 139: Take off “Qualitative” out of findings, the entire study is quantitative. - What does line 146 “input” mean? This is incomplete. - Line 143: authors should present a table on the main theme and sub themes in the manuscript and if they still want to maintain S3 Table, they can do so - Line 148, which researchers are you referring to? - Under introduction to findings lines 140-143, kindly give a summary of the findings that authors are going to present in detail in the subsequent write up. - Under Policies and Standard Operating Procedures: Authors are not presenting findings of the study, but their own conjecture. This section should be expunged. Authors should explain what the policies and guidelines are in the introduction section, so that they can later report on how they are being implemented in the results section based on the data they collected and NOT from the RESEARCHERS’ perspective. - Findings from lines 161 to 339, do not read like qualitative results but a listing of items. I am afraid, but the authors will need to do a more in depth analysis of the data to present actual findings that show the nuances, the depth of the issues studied, the themes among others. I strongly recommend that the authors should do a second level analysis of the critical or interesting issues report on. Also, authors interviewed different categories of respondents, but the different perspectives of these respondents are not reported in the different sections. The reader needs to read the similarities, contradictions among others from the different perspectives of the diverse groups of respondents. The sub headings are numerous and each contains very scanty information. I recommend that the authors should identify critical themes based on the objectives of the and do a thorough narration. - Sometimes the reader gets confused between a quote, a reference to policy document etc. I recommend that authors do proper quoting such as (a). indent quotes from the write up, (b). include quotation marks - Authors present quotations and attributes, without indicating the event such as whether it was in an IDI or a focus group discussion. In presenting observations, authors should do well to indicate where the observation took place, such as in a TB clinic, office etc and also include the date of the observation. | Recommendations incorporated, rephrased to remove numbers resembling quantitative and separating diverse group of respondents  The introductory statement corrected to match qualitative  The study is qualitative, Sociodemographics corrected to suit the right design  Sentence completed.  Table inserted  Amended, Table 3 with summary inserted. The policies and standard operating procedures were reported based on the observation as one of the tools used.  Indications on whether the quote is FGD,IDI or SSI captured to depict different categories views, most of them were in agreement though structured differently hence why there is mostly similarities.  Indications captured after every quote on results presentation, Dates also and place captured for observations. | Page 8-10  Page 9  Page 10-18  Page 19  Page 10-18  Page 10-18  Page 18-28  Page 19,21,26 |
| 13. Discussion  - Lines 347- 348: What does the following mean? “…… which we divided into various categories stretching across all framework levels.” Were the divisions made out of convenience or based on themes generated from the analysis of data? This should be clearly stated. - How do lines 380-382 support lines 377-380, considering that they are opposites, I thought that they contrast rather? - 380: why do authors state that “this was supported” instead of “this is supported by”. Also, what does “This” mean? - Line 390: what is DR Congo? - Line 386: what does ‘human resources’ mean? Be specific. - Lines 389-391: is this one study or several studies? The narration appears to be two different studies. - Line 398: several factors contributed to what? - In some of the paragraphs the authors refer to literature firs eg lines386-388. Besides the first paragraph that presents a summary of the findings the first sentence in the subsequent paragraphs should report on a finding first before presenting literature to compare, contrast among others. | Sentence construction amended to depict theme generated divisions.  Linking word changed to contrary and a justification provided  Tense changed to this is  DR Congo written in full  Sentence removed as it was duplicated on the last line of the stanza, human resource is captured as trained staff.  Its one multi country study and other two different studies, Sentence construction amended.  Sentence completed to capture poor community TB implementation.  Sentence removed as it was duplicated on the last lone of the stanza | Page 29  Page 30  Page 30  Page 31  Page 30  Page 30  Page 31  Page 30 |
| 14. Strength and Limitation  - The heading Strength and Limitation” is incomplete and should be in the plural - Line 436: Authors state: “Some limitations of this study are selection and social”, please elaborate on them - Authors report triangulating data and analysis as a strength, yet I did not find triangulation reported in data analysis. | Heading extended to include Strengths and Limitations of the study.  Extension added to include what brings about those bias  Indications(FGD,SSI,IDI) added after every quote on results presentation to depict data triangulation where there was variety of data from different sources | Page 32  Page 33  Page 18-28 |
| 15. Conclusion   Authors should present two to three strong recommendations based on their findings in the concluding section  Consent for publication   - How was confidentiality and anonymity in data collection ensured | One more recommendation added.  Written informed consent was obtained from each participant before the interviews/discussions. | Page 33  Page 8 |
