# Supplementary material for: Potential facilitators and inhibitors to the implementation and sustainability of the community-based tuberculosis care interventions. A case study from Moshupa, Botswana
Source: PLoS One. 2023 Aug 10;18(8):e0290010. doi: 10.1371/journal.pone.0290010 (PMC10414663; doi:10.1371/journal.pone.0290010)
Supplement: Attachment — Submitted filename: Response to reviewers.docx [file pone.0290010.s007.docx]

The following are the editors’ comments/recommended changes to the Manuscript, authors responses and page number where changes were made presented in Tabular form.

| ***Editor’s comments/Recommended Changes*** | ***Authors Response*** | ***Changes Page Number*** |
| --- | --- | --- |
| 1. The authors need to edit the entire manuscript for wrong tenses and missing articles especially the sections in track changes | Tenses corrected | Entire paper |
| 2. Page 18 line 219 has an incomplete sentence | Sentence completed | Page 20 |
| 3. The quotations are not aligned, they are not indented, and they do not have quotation marks. Also, the authors do not use the appropriate formatting for quotations. I recommend that they refer to the AAA style guide to carry out the necessary revisions (American Anthropological Association. AAA Style Guide 2009, 2009. Available from: https://socioanthro.uoguelph.ca/sites/default/files/page-files/American-Anthropological-Association-Style-Guide.pdf.). | Quotations inserted and quotes indented | Page 19-31 |
| 4. The presentation of the findings is very poorly done, the authors do not make efforts to interpret the results before citing the quotations. I urge the authors to read a few well written qualitative articles to improve upon the presentation of the results. | Results interpretation addressed | Page 19-31 |
| 5. The in-text citations within the information in tracks are not not properly done. | Change incorporated |  |
| 6. The tables within the manuscript are labelled as S1 Table etcetera? | The journal Submission Guidelines requires they be labelled that way | Page 4  Page 9  Page 10 |
