# Supplementary material for: Potential facilitators and inhibitors to the implementation and sustainability of the community-based tuberculosis care interventions. A case study from Moshupa, Botswana
Source: PLoS One. 2023 Aug 10;18(8):e0290010. doi: 10.1371/journal.pone.0290010 (PMC10414663; doi:10.1371/journal.pone.0290010)
Supplement: Attachment — Submitted filename: PONE-D-22-32443-Reviewer comments.pdf [file pone.0290010.s005.pdf]

Potential facilitators and inhibitors to the implementation and sustainability of the community-based tuberculosis care interventions. A case study from Moshupa district, Botswana

Reviewer comments

This paper is a potential contribution to the literature on community-based support for the management of tuberculosis in Botswana. It is based on an apparently failed community TB care (CTBC) program in Botswana. The study used a qualitative approach and all the elements of standard manuscript have been included. The quality of writing is acceptable although thorough proofreading will be needed before publication.

Major concern

- The description of the methods section is not detailed enough to permit replication of the study without recourse to the authors. The design is described in a maze of imprecise lexicon –"*Interpretive 7 paradigm based on relativist ontology and subjectivist epistemology along with adductive research*". This should be revised. As nebulous are these are, there is very little reflection of their application in the analysis and reporting of the study.
- Important details like who conducted the interviews, where they were conducted, the nature of interview guide, how was the FGD organized? Was it one FGD with 8 community leaders?
- It is reported that clinic-based observations were conducted. There is however no information on how they were conducted. By whom? When and how? Was it with a standard tool? How was analysis of that data conducted?

Other concerns

- The descriptive results in the opening paragraph of results is not comprehensive enough. The authors need to detail out the overall picture before going into the description by gender and age. And for each of the approaches to data collection, the authors need to be detailed in the characteristics of the participants
- The results should also present a triangulation of information and not just a topic by topic representation with quotes. It is out of the triangulation of information that new understanding or theory emerges to add something new to the literature.
- While it is well to present the Botswana program as having failed and needing to be researched, it is important for evidence to that effect to be presented. The authors need to provide data to back the claim of "low treatment outcome (**this statement is unclear**)" and "high case notification"? There should also be an attempt to link the above shortcomings to the delivery of Community TB Care.
